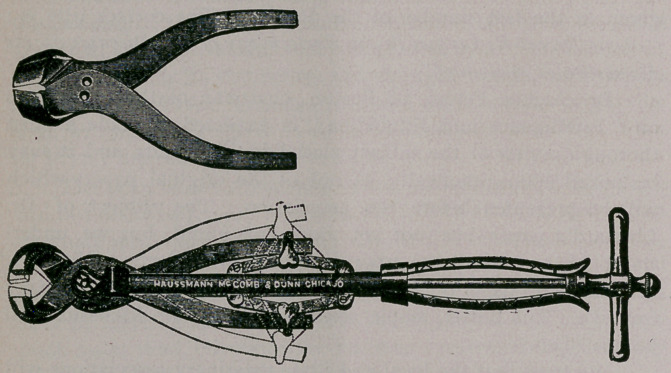# Molar Extractor and Cutter

**Published:** 1890-06

**Authors:** 


### MOLAR EXTRACTOR AND CUTTER.

WE are indebted to Messrs. Haussman, McComb & Dunn, of Chicago, for a new instrument for extracting and cutting the molar teeth of the horse and cow.

This instrument consists of a central stalk, through which runs a powerful screw. The extractors or cutters, which are interchangeable, are annexed to the stalk and act on a pin hinge at the center of an **X**, which they form. The shorter arms of the **X** have roughened teeth or cutting edges, while the longer arms, which give great power, are moved by a double set of bars which are acted upon by the screw.

This instrument has the advantage of great power and moderate bulk, which latter makes it more convenient than some of the older French and English instruments.

### JOINT MEETING OF THE ILLINOIS AND INDIANA VETERINARY MEDICAL ASSOCIATIONS.

A MEETING of the above associations will be held at Terre Haute, Ind., on June 4th and 5th, 1890.

The following programme promises a most instructive and attractive meeting: On the first day, evening session, there will be an "Address of Welcome," by F. C. Donaldson; an address, by Eugene V. Debs; a paper on the "Pathology of Azoturia as Suggested by its History and Symptoms," by Dr. W. L. Williams, of Bloomington, Ill., and a paper on the "Surgery of Fistula," by Dr. R. C. Mylne.

June 5th the association will visit points of interest, including fair grounds and important stock farms. In the afternoon a recess will be taken for those desiring to attend races, in the evening there will be a paper on "Pneumonia," by Dr. G. W. Buckner; a paper by Drs. H. R. Macaulay and A. J. Thompson; a paper on "Swine Plague," by Dr. F. S. Billings. Other papers, the subjects of which have not yet been received, will be presented.

A very large attendance is expected. Communications should be addressed to Dr. E. M. Knowles, president, Terre Haute, Ind., or the secretary, Dr. H. R. Macaulay, Indianapolis, Ind.

A full report of the meeting will be given in the next number of the Journal.

## Figures and Tables

**Figure f1:**